# Teaching Future Nurses to Support Older Clients’ Autonomy in Activities of Daily Living

**DOI:** 10.1093/geroni/igaf122.2695

**Published:** 2025-12-31

**Authors:** Marjolein Knibbeler, Stan Vluggen, Erik van Rossum, Sandra Zwakhalen, Petra Erkens

**Affiliations:** Maastricht University, Maastricht, Limburg, Netherlands; Zuyd University of Applied Sciences, Heerlen, Limburg, Netherlands; Zuyd University of Applied Sciences, Heerlen, Limburg, Netherlands; Maastricht University, Maastricht, Limburg, Netherlands; Maastricht University, Maastricht, Limburg, Netherlands

## Abstract

Supporting older clients’ autonomy in daily care is crucial for dignity and quality of life. However, research indicates that nurses often provide suboptimal autonomy-support, and little is known about how future nurses learn this skill during their education. A qualitative study examined both theoretical and practical learning. The theoretical part involved a document study of Dutch nursing curricula and teaching materials to assess how autonomy-supportive behaviour is addressed. Additionally, 20 semi-structured interviews with teachers and students explored their perspectives on autonomy in education. Practical learning was studied through 25 observations of student-supervisor interactions during activities of daily living. Furthermore, 22 follow-up interviews with students and supervisors provided deeper insights into their experiences. Findings indicate that while curricula mention autonomy, they provide little practical guidance. Teachers acknowledge its importance but rely largely on personal experience rather than structured educational methods. Students report learning autonomy-supportive behaviour primarily through hands-on training. Observations confirm that autonomy is more emphasized in practice, yet supervisors’ approaches vary widely. Some prioritize autonomy, fostering client independence, while others emphasize efficiency, sometimes at the cost of client-autonomy. Given the evolving needs of an aging population, rethinking nursing education on autonomy is essential. Strengthening collaboration between teachers and practical supervisors can create a more cohesive learning environment. Additionally, integrating innovative teaching methods, such as simulation-based learning and case discussions, can bridge the gap between theoretical and practical education. Enhancing nursing education will ensure future nurses are well-equipped to support autonomy, ultimately improving person-centered elderly care.

